# Low-Dose Aspartame Consumption Differentially Affects Gut Microbiota-Host Metabolic Interactions in the Diet-Induced Obese Rat

**DOI:** 10.1371/journal.pone.0109841

**Published:** 2014-10-14

**Authors:** Marie S. A. Palmnäs, Theresa E. Cowan, Marc R. Bomhof, Juliet Su, Raylene A. Reimer, Hans J. Vogel, Dustin S. Hittel, Jane Shearer

**Affiliations:** 1 Department of Biochemistry and Molecular Biology, University of Calgary, Calgary, Alberta, Canada; 2 Department of Biological Sciences, University of Calgary, Calgary, Alberta, Canada; 3 Faculty of Kinesiology, University of Calgary, Calgary, Alberta, Canada; University of East Anglia, United Kingdom

## Abstract

Aspartame consumption is implicated in the development of obesity and metabolic disease despite the intention of limiting caloric intake. The mechanisms responsible for this association remain unclear, but may involve circulating metabolites and the gut microbiota. Aims were to examine the impact of chronic low-dose aspartame consumption on anthropometric, metabolic and microbial parameters in a diet-induced obese model. Male Sprague-Dawley rats were randomized into a standard chow diet (CH, 12% kcal fat) or high fat (HF, 60% kcal fat) and further into *ad libitum* water control (W) or low-dose aspartame (A, 5–7 mg/kg/d in drinking water) treatments for 8 week (n = 10–12 animals/treatment). Animals on aspartame consumed fewer calories, gained less weight and had a more favorable body composition when challenged with HF compared to animals consuming water. Despite this, aspartame elevated fasting glucose levels and an insulin tolerance test showed aspartame to impair insulin-stimulated glucose disposal in both CH and HF, independently of body composition. Fecal analysis of gut bacterial composition showed aspartame to increase total bacteria, the abundance of *Enterobacteriaceae* and *Clostridium leptum*. An interaction between HF and aspartame was also observed for *Roseburia* ssp wherein HF-A was higher than HF-W (P<0.05). Within HF, aspartame attenuated the typical HF-induced increase in the Firmicutes:Bacteroidetes ratio. Serum metabolomics analysis revealed aspartame to be rapidly metabolized and to be associated with elevations in the short chain fatty acid propionate, a bacterial end product and highly gluconeogenic substrate, potentially explaining its negative affects on insulin tolerance. How aspartame influences gut microbial composition and the implications of these changes on the development of metabolic disease require further investigation.

## Introduction

Regular consumption of artificially sweetened soft drinks is associated with disorders of the metabolic syndrome, including abdominal obesity, insulin resistance and/or impaired glucose tolerance, dyslipidemia and high blood pressure [1]–[3]. In particular, daily diet soda consumption (primarily sweetened with N-a-L-aspartyl-L-phenylalanine methyl ester, aspartame, APM), is reported to increase the relative risk of type 2 diabetes and the metabolic syndrome by 67% and 36% respectively [3]. Given this data, and the presence of APM in over 6000 food products, there is a need to understand the potential role of APM sweetened products in the development and maintenance of metabolic disease [4].

Emerging evidence on the gut microbiome suggests that metabolic diseases, such as type 2 diabetes, are associated with an altered gut microbiota profile [5], [6]. The gut microbiome plays an important role in metabolism and caloric extraction from dietary sources. It is highly complex and one of the most diverse ecosystems, with over 50 phyla identified [7], [8]. Alterations in the proportions of the two phyla that make up ∼90% of the human gut microbiome, Firmicutes and Bacteroidetes, have been linked to obesity, type 2 diabetes and systemic inflammation [8]–[10] with the majority of studies reporting increases in the abundance of Firmicutes and reductions in Bacteroidetes compared to lean individuals [5]–[7], [11]. Compositional and functional changes in the microbiome are also manifested as alterations of metabolite concentrations in the blood. Microbial metabolites appearing in serum consist of metabolic intermediates, organic acids and bacterial fermentation end products including the short chain fatty acids (SCFA) [12]–[14].

Aims of the present study were to examine the interaction of chronic low-dose APM on anthropometric, metabolic, metabolomic and gut microbiota profiles. As observational data in humans cannot show causality, we examined an animal model where the direct effects of APM on metabolism could be established. Specifically, we investigated the impact of low-dose APM (5–7 mg/kg/d, equivalent to consuming 2–3 cans of diet soda per day for the average US male and female (∼89 kg and 76 kg respectively)) [14], a dose well below the upper daily-recommended intake of 40–50 mg/kg/d [4] in the diet-induced obese, Sprague-Dawley rat. If APM alters the gut microbiota, and in turn the serum metabolome, such changes would likely appear in this well-characterized model and could provide insight into the relationship between this artificial sweetener and the development of metabolic disease.

## Methods

### Animal experiments

Experimental procedures were performed under the ethical standards approved by the University of Calgary Animal Care and Use Committee (AC11-0016) as well as guidelines established by the Canadian Council on Animal Care. Male Sprague-Dawley rats (n = 44, Charles River, Wilmington, MA) were housed individually in a 12 h light/dark cycle. Animals were randomized into two dietary groups; chow (CH 12% kcal fat) (Lab Diet 5001, St. Louis, MO) or high fat (HF 60% kcal fat) (Open Source Diets, Research Diet # D12492, New Brunswick) for two weeks and then randomly assigned fluid treatment (i.e. water or APM). APM was directly added to drinking water (60 mg/L, Merisant Company, Chicago, IL). All animals, had access to food and fluid *ad libitum* for an additional 8 week prior to sacrifice. This resulted in four treatment groups (n = 10–12 per treatment); chow water (CHW), high fat water (HFW), chow aspartame (CHA) and high fat aspartame (HFA). Data from CHW and HFW were part of a shared control group that has been previously published [15].

### Weight gain and body composition

Animals were weighed weekly for 10 weeks. Food and fluid intake was measured during week 7 of the diet. Dual energy x-ray absorptiometry with small animal software (Hologic QDR 4500, Hologic, Inc., Bedford, MA) was used to determine lean mass and fat mass, as well as bone mineral density, during week 10, prior to sacrifice, as previously described [16]. On the day of sacrifice, animals were anesthetized with isoflurane (2-chloro-2-(difluoromethoxy)-1,1,1-trifluoro-ethane)(Sigma Aldrich, Oakville, ON, Canada). Following anesthesia, blood samples were rapidly collected on anesthetized animals by cardiac puncture through the chest wall. After blood collection, the liver was rapidly excised, rinsed in saline to remove excess blood, freeze-clamped in liquid nitrogen, and kept frozen at −80°C until further analysis. Blood samples were aliquoted into two tubes for serum and plasma collection. In the first tube, blood clotted (no additives) for 20 min (4°C) and serum was isolated via centrifugation for metabolomics analyses. The second aliquot was placed in a chilled tube containing ethylenediaminetetraacetic acid (EDTA), diprotinin-A (0.034 mg/ml blood; (MP Biomedicals, Irvine, CA)), Sigma protease inhibitor (1 mg/ml blood; Sigma Aldrich, Oakville, ON, Canada) and Roche Pefabloc (1 mg/ml of blood; Roche, Mississauga, ON, Canada). This sample was used for plasma measures including insulin, gastric inhibitory polypeptide (GIP) and free fatty acids. Both serum and plasma samples were stored at −80°C until analysis.

### Biochemical analyses

Plasma free fatty acids were quantified using a HR Series NEFA-HR kit (Wako Chuo-Ku, Osaka, Japan). Plasma insulin and GIP were measured using a Milliplex Map Kit Rat Gut Hormone Panel 96 well plate assay for Insulin and GIP (EMD Millipore Corporation, Billerica, MA). Liver triglycerides were measured using Triglyceride (GPO) (Liquid) Reagent Set (Pointe Scientific Inc., Canton MI) as per manufacturers instructions.

### Oral glucose and insulin tolerance tests

During week 8, animals were fasted for 8 h overnight prior to an oral glucose tolerance test (OGTT). Animals were weighed and 100 µL of blood was collected via tail clip (0.5 mm tip of tail) in conscious rats. Fasting blood glucose concentrations were measured using a standard blood glucose monitor (BD BioSciences, Franklin Lakes, NJ). Following the initial blood glucose measurement, rats received an oral glucose load (2 mg/kg body weight) and subsequent blood samples were taken at 15, 30, 60, 90 and 120 minutes and blood glucose concentration determined immediately. Following a one-week washout period, animals were again fasted and an insulin tolerance test (ITT) was administered. This was done by injecting Humulin R insulin (Eli Lilly Canada, Toronto ON) diluted 100X with saline into the intraperitoneal cavity (0.75 U/kg). Blood samples were obtained and glucose measured immediately at time points identical to the OGTT.

### Serum metabolomics analysis

Metabolomics analysis of serum samples was performed by proton nuclear magnetic resonance spectroscopy (^1^H NMR) as previously described with minor modifications [17], [18]. Samples were coded with sample ID and prepared, analyzed and profiled in a randomized order. Briefly, ^1^H NMR spectra were acquired using the standard pulse program (prnoesy1d) on a Bruker Avance 600 spectrometer (600.22 MHz, 297K 5 mm TXI Probe). Initial processing was performed for the first sample in each batch using TopSpin software. Each sample was then individually processed using Chenomx NMR Suite 7.5 software (Chenomx Inc., Edmonton, Canada). Targeted profiling was performed using the NMR Suite profiling module, applying the Chenomx library. The Human Metabolome Database, accessible at http://www.hmdb.ca, aided metabolite identification. Two-dimensional total correlation spectroscopy and heteronuclear single quantum coherence spectroscopy spectra were performed on the last sample of the batch for metabolite validation.

### DNA extraction and qRT-PCR analysis

Fresh fecal samples were collected at 10 weeks. Samples were stored at −80°C until further analysis. DNA was extracted from 250 mg of fecal matter using the FastDNA Spin Kit for Feces (MP Biomedicals, LLC, Solon, OH). DNA concentrations were quantified using the Nanodrop 2000 (Thermo Fisher Scientific Inc., Asheville, NC), diluted to 4 ng/µl, then stored at −20°C until analysis. Amplification and detection were conducted in 96 well plates with SYBR Green 2× qPCR Master Mix (BioRad). Samples were analyzed in duplicate with a final volume of 25 µl containing 0.3 µM primer and 20 ng template gDNA. Group specific primers have been previously published [19]. The 16S rRNA gene copies value was calculated according the following webpage: http://cels.uri.edu/gsc/cndna.html using average genome sizes. Standard curves were normalized to the copy number of the 16S rRNA gene obtained from the following: http://rrndb.mmg.msu.edu/index.php.

### Statistical analysis

SigmaStat version 3.5 (SYSTAT, Chicago, IL) was used for parametric statistical analyses of the biometric and the microbiota data. Where appropriate, microbiota data is reported on a log scale and shown as mean ± SE. Differences between the four dietary groups were determined by a two-way ANOVA, followed by a Student-Newman-Keuls post-hoc test (*p*<0.05). A two-tiered method was employed to analyze metabolomics data as previously described [18], [20]. Initial analysis consisted of multivariate statistical analysis. Normalized ^1^H NMR data was imported into SIMCA-P+ software (version 12.01, Umetrics AB, Umeå, Sweden), performing initial mean centering and unit variance scaling. Unsupervised principle component analysis was conducted to identify and visualize initial grouping of the data and potential outliers (i.e. samples outside the 95% CI). Graphical representation of these results is shown in **Figures S1, S2**. Individual serum metabolites were then analyzed by a two-way ANOVA corrected for a false discovery rate of 20% according to Benjamini and Hochberg [21].

## Results

### Animal characteristics

Anthropometric, metabolic as well as food and fluid consumption data are shown in **Table 1**. HF animals were obese with an increase in body fat compared to their CH fed counterparts (*p*<0.05). No differences in bone mineral density were found between groups (data not shown, *p*>0.05). Liver triglycerides were elevated with HF (*p*<0.05) but not APM treatment. CHA and HFA consumed 17 and 25% less energy (kcal), but more fluid compared to their respective controls (*p*<0.05). Fasting blood glucose levels were elevated in both APM groups (*p*<0.05). Likewise, plasma insulin levels increased with HF (CHW vs. HFW), however, within HF, APM consumption normalized plasma insulin levels to those observed for CH (*p*<0.05). The combination of HF and APM also resulted in increases in circulating plasma free fatty acid levels (*p*<0.05). Analysis of the insulin-related gut hormone GIP showed no differences between treatments (*p*>0.05).

**Table 1:** Characteristics of experimental animals.

	Chow		High Fat	
	Water	Aspartame	Water	Aspartame
**Final weight (g)**	488±^5.5^	453±13.2	641±17.0*	533±22.5*^†^
**Body Fat (%)**	7.1±0.5	11.9±1.0^†^	28.7±1.5*	21.0±1.8*^†^
**Liver triglycerides (mM)**	17.7±1.6	16.8±0.9	47.1±5.9*	43.3±4.6*
**Food consumption (kcal/day)**	132.1±2.3	109.5±8.0^†^	152.3±8.9*	113.1±5.1^†^
**Fluid consumption (ml/day)**	42.4±1.3	47.7±3.9^†^	27.7±1.4*	38.8±2.4^†^
**Sweetener consumption (mg/kg/day)**	----	7.0±0.5	----	4.9±0.3*
**Fasting blood glucose (mM)**	5.4±0.2	6.9±0.4^†^	5.6±0.2	7.4±0.6^†^
**Plasma Insulin (pmol/l)**	143.1±13.8	143.8±27.5	197.4±29.3	123.7±13.7^†^
**Plasma free fatty acids (mM)**	0.24±0.03	0.25±0.02	0.35±0.05	0.48±0.06*^†^
**Plasma GIP (pmol/l)**	12.2±2.8	8.4±2.4	10.3±1.9	11.3±2.5

Food and fluid consumption were recorded during week 7 of the diet. Glucose was measured in the fasted state, all others including plasma free fatty acids, liver triglycerides and plasma insulin were measured from samples taken at sacrifice (non-fasting). All data includes n = 9–12 animals/group, data is represented as mean ± SE. *p<0.05 for diet (chow vs. high fat) within fluid treatments (water, aspartame). ^†^ *p*<0.05 for fluid (water vs. aspartame) within diet (chow, high fat). Data from the water controls (chow, high fat) were part of a shared control group that has been previously published [15]. Permission to reuse the data in this table was obtained from Elsevier.

### Oral glucose and insulin tolerance tests

**Figure 1:**
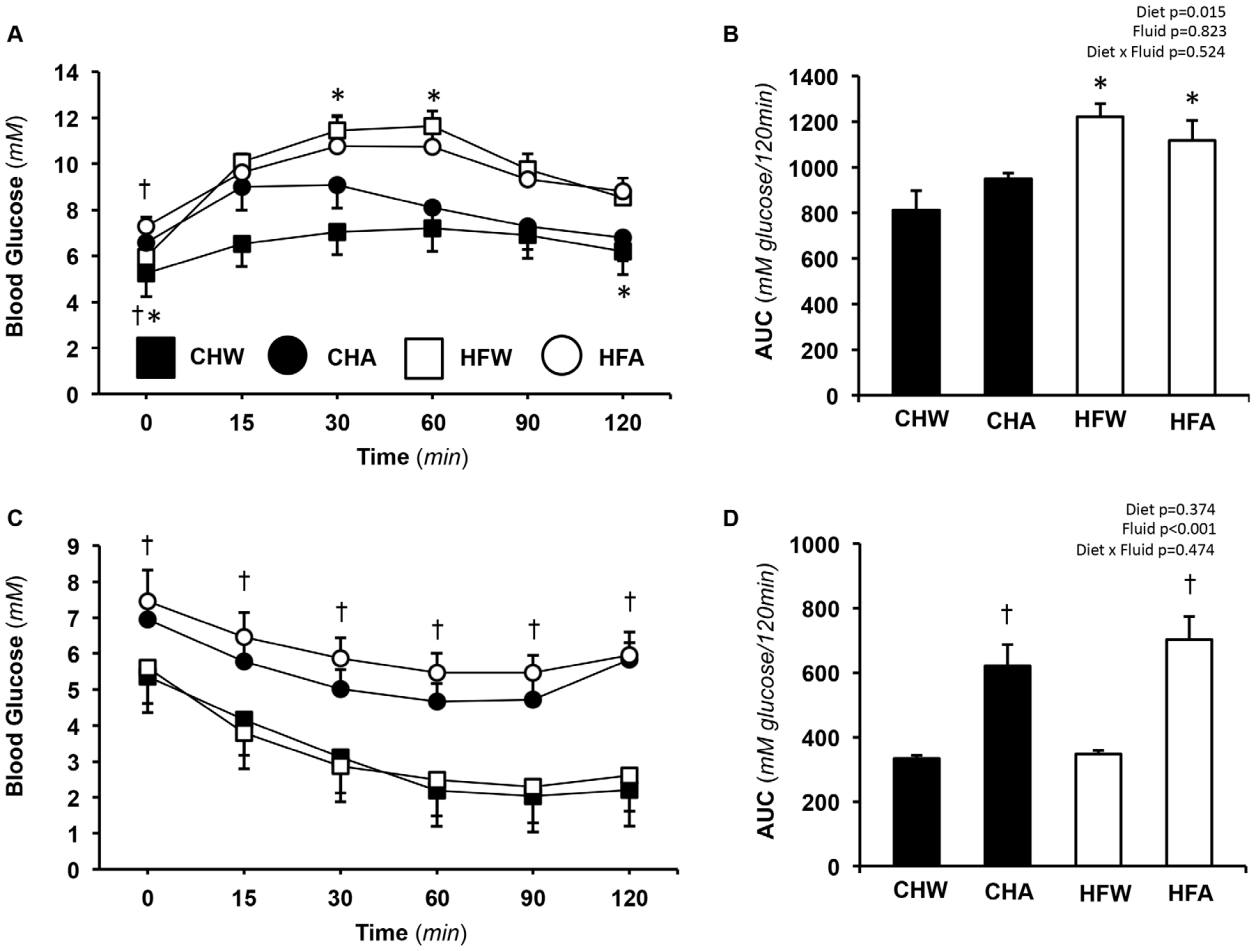
Measures of glucose tolerance and insulin sensitivity. **A**. Blood glucose from the oral glucose tolerance test (OGTT) from 0–120 min. **B**. Total area under the curve for OGTT over 120 min. **C**. Blood glucose following an insulin tolerance test (ITT). **D**. Total area under the curve for the ITT over 120 min. Data represents means ± SE, n = 9–12 per treatment. † p<0.05 for fluid (water vs.aspartame) within diet (chow, high fat). Statistics (*p* values) for area under the curve data (diet, fluid) and their interactions are also shown, p<0.05 being considered significant. Data from the water controls (chow, high fat) were part of a shared control group that has been previously published [15]. Permission to reuse the data in this figure was obtained from Elsevier. Abbreviations are as follows; CHW, chow water; CHA, chow aspartame; HFW, high fat water; HFA, high fat aspartame.

Results of the OGTT administered on week 8 of the diet are shown in **Figure 1A and 1B**.

Fasting blood glucose was higher in APM versus control rats in both CH and HF (*p*<0.05) (**Table 1**). There was a significant main effect of diet for blood glucose AUC wherein HF was higher than CH (*p*<0.05). No main effect of APM on blood glucose AUC was observed (*p*>0.05).

Following a one-week wash-out period, the ITT was administered on week 9. Results are shown in **Figure 1C and 1D**. For the ITT, both APM groups started with elevated blood glucose levels that persisted for the duration of the test (*p*<0.05). Independently, fluid but not diet (*p*<0.05), affected glucose disappearance during the ITT with CHA and HFA exhibiting impaired insulin-stimulated glucose disposal compared to their respective water controls (CHW and HFW) (*p*<0.05).

### Gut microbiota analysis

Results of gut microbiota analysis are shown in **Table 2**. As expected, HF feeding alone (CHW vs. HFW) perturbed the gut microbiota with increases in total bacteria, Firmicutes, and *Clostridium Cluster C XI* (*p*<0.05). HF also decreased the abundance of *Bacteroides/Prevotella* spp. (*p*<0.05). Within diets, few differences between the CH treatment groups (CHW vs. CHA) were observed with the exception of *Clostridium leptum*, which was higher in the CHA group versus CHW (*p*<0.05). This finding was consistent for HF as well where HFA had higher *Clostridium leptum* compared to HFW (*p*<0.05). Diet affected *Bifidobacterium* spp. with greater abundance in the HF versus CH groups. Total bacteria, *Enterobacteriaceae* and *Roseburia* spp. were all influenced by the interaction of diet and APM where the HFA treatment resulted in the highest abundance of each bacterial group.

**Table 2:** Gut microbiota composition of fresh fecal samples collected on week 10 of the diet and fluid (water or aspartame, APM) treatments.

	Chow		High Fat		*p* values		
Bacteria	Water	Aspartame	Water	Aspartame	Diet	Aspartame	Diet x APM
Total bacteria	7.91±0.056	7.74±0.026	7.99±0.133	8.01±0.080*^†^	0.012	<0.001	0.005
*Bacteroides/Prevotella* spp.	7.47±0.092	7.25±0.142	6.46±0.076*	6.62±0.069*	<0.001	0.244	0.153
*Bifidobacterium* spp.	5.51±0.199	4.55±0.114	6.57±0.313*	5.97±0.316^†^	0.011	0.062	0.126
*Enterobacteriaceae*	4.08±0.084	4.72±0.117	4.56±0.108	5.26±0.112*^†^	<0.001	<0.001	0.003
Firmicutes	7.69±0.078	7.55±0.048	7.94±0.145*	7.98±0.084	0.014	0.263	0.477
*Lactobacillus* spp.	7.32±0.072	6.94±0.095	7.45±0.194	7.08±0.105	0.489	0.212	0.908
*Clostridium leptum*	6.51±0.042	7.16±0.110^†^	6.93±0.104	7.24±0.159^†^	0.088	<0.001	0.637
*Clostridium coccoides*	6.69±0.065	6.66±0.060	6.68±0.048	6.86±0.060	0.137	0.205	0.095
*Clostridium* cluster (CI)	5.64±0.087	5.72±0.270	6.12±0.185	5.96±0.167	0.103	0.672	0.519
*Clostridium* cluster (CXI)	7.02±0.126	6.44±0.152	7.96±0.187*	7.58±0.049	0.004	0.115	0.238
*Roseburia* spp.	6.88±0.157	6.69±0.253	6.42±0.132	7.71±0.344*^†^	0.434	0.135	0.022

Data represent Log 16S rRNA gene copies/20 ng total genomic DNA, mean ± SE, n = 9–12 per treatment. *p<0.05 for diet (chow vs. high fat) within fluid treatments (water, aspartame). ^†^ *p*<0.05 for fluid (water vs. aspartame) within diet (chow, high fat). Data from the water controls (chow, high fat) were part of a shared control group that has been previously published [15]. Permission to reuse the data in this table was obtained from Elsevier.

**Figure 2:**
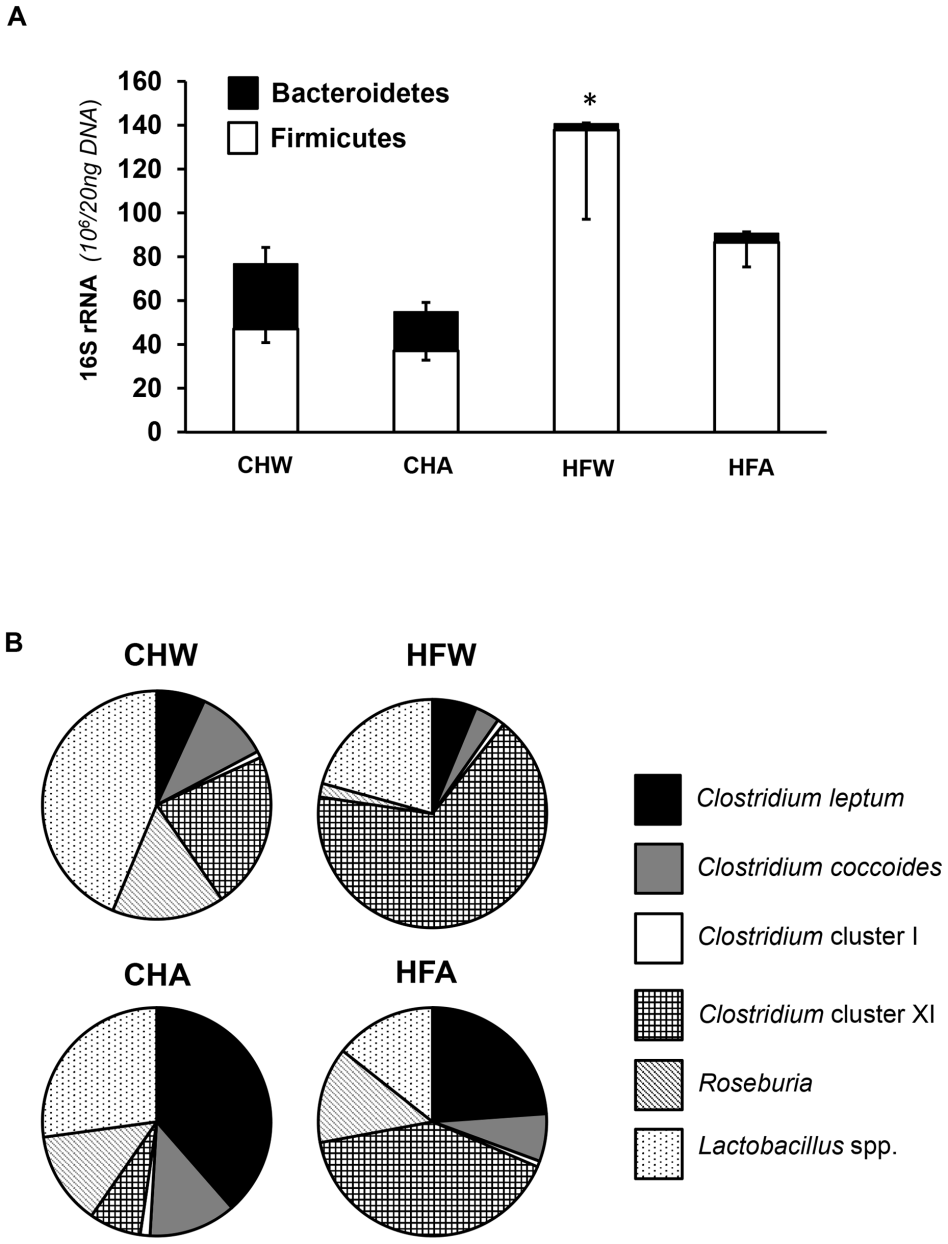
Gut microbiota analyses of diet and fluid treatments. **A**. Graphical representation of the absolute changes in the Firmicutes:Bacteroidetes ratio in fresh fecal matter resulting from dietary (chow or high fat) or fluid (water or aspartame) treatment. HFW has elevated levels in comparison to the other groups, with the data representing absolute number (10^6^) of 16S rRNA gene copies per 20 ng DNA. The numerical value above each bar represents the Firmicutes:Bacteroidetes value. **B**. Relative bacterial abundance within the Firmicutes phyla. Data is based on 16S rRNA gene copies (10^6^/20 ng DNA), on a relative (100%) scale. Consistent with the absolute results (**Table 2**), APM treatment within HF (HFA) increased the relative proportion of *Clostridium leptum* and attenuated high fat- increased in *Clostridium* cluster XI. Data from the water controls (chow, high fat) were part of a shared control group that has been previously published [15]. Permission to reuse the data in this figure was obtained from Elsevier. Abbreviations are as follows: CHW, chow water; CHA, chow aspartame; HFW, high fat water; HFA, high fat aspartame. Data represents means ± SE, n = 9–12 per treatment.

As an elevated Firmicutes to Bacteroidetes ratio has been previously documented with obesity, this ratio was also examined. When expressed on an absolute scale, HF resulted in a decrease in Bacteroidetes and an increase in Firmicutes (**Figure 2A**). Within HF, APM treatment (HFA) attenuated the increase in Firmicutes, with little effect on Bacteroidetes. To further examine changes in these phyla, the relative proportions of bacteria within the Firmicutes phyla were plotted on a relative scale (100% total) as shown in **Figure 2B**. Consistent with **Table 2** results, APM treatment within HF (HFA) increased the relative proportion of *Clostridium leptum* and attenuated HF-induced increases in *Clostridium* cluster XI.

### Serum metabolomic analysis

**Figure 3:**
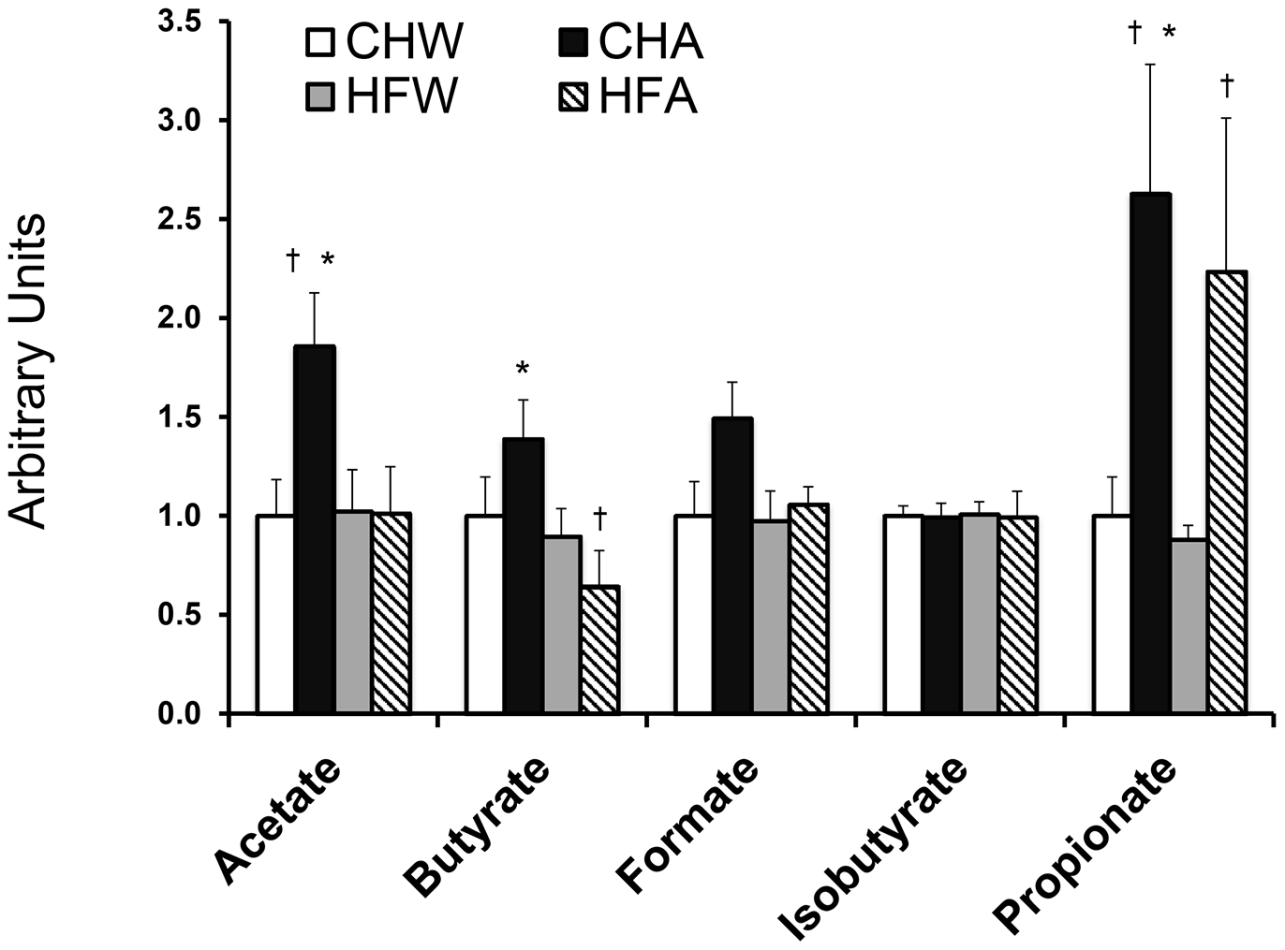
Short-chain fatty acid concentrations from serum metabolomics analysis. Relative changes in the serum short chain fatty acids using ^1^H NMR spectroscopy. Data represents means ± SE, n = 9–12 per treatment. Data are shown relative to chow water, set as a value of 1.0. * p<0.05 for diet (chow vs. high fat) within fluid treatments (water, aspartame); † p<0.05 for fluid (water vs. aspartame) within diet (chow, high fat).

Serum metabolites changing in response to diet, fluid and the interaction of the two treatments are shown in **Table 3**. APM breakdown products including aspartate, methanol and phenylalanine were not elevated in the APM consuming animals, indicating rapid metabolism of the sweetener. APM treatment led to changes in nine serum metabolites including lysine, serine, glycine, propionate, creatine, 3-hydroxybutyrate, methanol, glycerol and urea. As previous reports have documented differential effects of APM in lean and obese subjects [22], the interaction between diet and fluid was also examined. Creatine, acetate, butyrate, myo-inositol and dimethyl sulfone were all affected by the interaction of diet and fluid. Of the serum metabolites detected, the SCFA (acetate, butyrate, formate, isobutyrate and propionate) predominated and were of particular interest because of their bacterial origin (**Figure 3**). APM increased levels of acetate and butyrate in CH groups, while formate and isobutyrate remain unchanged. APM resulted in elevated circulating propionate levels by ∼2.5 fold in both CHA and HFA compared to CHW and HFW respectively (p<0.05). Results of multivariate statistical analysis of the serum metabolomic profiles are shown in **Figure S1, S2**, visualizing the impact of both diet and APM treatments. The major source of variation in the dataset was explained by three principal components, with a cumulatively explained variance of 41.1%.

**Table 3:** List of detected serum metabolites identified with ^1^H NMR analysis.

Serum Metabolite	Diet	Fluid	Diet-Fluid Interaction
***Amino Acids*** Alanine	CH>HF	-	-
Arginine	-	-	-
Asparagine	-	-	-
Betaine	CH>HF	A>W	-
Aspartate	<LLOD	<LLOD	<LLOD
Choline	-	-	-
Creatine	CH>HF	A>W	CHA>HFA and CHA>CHW
Glutamate	-	-	-
Glutamine	-	-	-
Glycine	CH>HF	A>W	-
Histidine	-	-	-
Isoleucine	-	-	-
Leucine	-	-	-
Lysine	-	A>W	-
Methionine	CH>HF	-	-
Ornithine	-	-	-
Phenylalanine	-	-	-
Proline	-	-	-
Serine	-	A>W	-
Taurine	-	-	-
Threonine	-	-	-
Tyrosine	-	-	-
Valine	-	-	-
***Short-Chain Fatty Acids*** Acetate	-	-	CHA>HFA and CHA>CHW
Butyrate	CH>HF	-	CHA>HFA and HFW>HFA
Formate	-	-	-
Propionate	-	A>W	-
Isobutyrate	-	-	-
***Ketone Bodies*** Acetoacetate	-	-	-
Acetone	-	-	-
3-Hydroxybutyrate	-	A>W	-
***Alcohols*** Methanol	-	W>A	-
Myo-inositol	-	-	CHA>HFA and CHA>CHW
Glycerol	-	W>A	-
Propylene glycol	-	-	-
***Other Metabolites*** 2-Hydroxybutyrate	-	-	-
2-Hydroxyisobutyrate	-	-	-
Carnitine	-	-	-
Citrate	-	-	-
Creatine phosphate	-	-	-
Creatinine	-	-	-
Dimethyl sulfone	CH>HF	-	CHA>HFA and HFW>HFA
Histamine	-	-	-
Lactate	CH>HF	-	-
N-Isovaleroylglycine	-	-	-
O-Acetylcarnitine	CH>HF	-	-
O-Phosphocholine	-	-	-
Pyruvate	-	-	-
Urea	-	W>A	-

Notation indicates a significant difference (p<0.05) and the direction of change while ‘-’ indicates no difference detected (p>0.05). Statistical interactions between diet x fluid are also shown. Analysis was based upon a two-tiered method as previously described [18], [20]. Initial analysis consisted of multivariate statistical analysis, confirmed by a two-way ANOVA corrected for a false discovery rate of 20% according to Benjamin and Hochberg [21]. Data represent n = 9–12 per treatment. Abbreviations are as follows: CH, chow; HF, high fat; W, water; A, aspartame.

## Discussion

There is continuing controversy over the impact of chronic APM consumption on the risk and development of obesity, type 2 diabetes and the metabolic syndrome [23], [24]. Population-based studies have found both associations [1]–[3], and no associations [25] between diet soft drink intake and metabolic disease. These disparate results may be ascribed to the difficulty of controlling confounding variables in a human population; for example, obese and diabetic individuals generally consume more diet soft drinks and APM containing products than non-diabetics [26]. For this reason, we chose to examine the impact of chronic, low-dose APM consumption in a lean and diet-induced obese animal model where confounding variables could be strictly controlled.

Results of the present study show APM to differentially affect measures associated with metabolic disease. Major findings were as follows: i) APM lowered net energy consumption and body mass in both CH and HF. In HF, APM resulted in lower body fat percentage as well as a decline in plasma insulin levels; ii) APM consumption was associated with fasting hyperglycemia and impaired insulin tolerance in both CH and HF; iii) APM resulted in distinctive changes in the gut microbiota including increases in *Enterobacteriaceae* and *Clostridium leptum*. Within HF, APM attenuated typical HF-induced increases in the Firmicutes:Bacteroidetes ratio and resulted in an elevation in *Roseburia* ssp.; and iv) APM increased serum propionate, a SCFA of bacterial origin. The effects were observed despite the small quantities of APM consumed (∼5–7 mg/kg/d), as previously mentioned equivalent to an approximate human consumption of 2–3 diet soft drinks per day for 8 weeks. These results show APM, even at very low doses, to have multiple and complex effects on metabolic health.

There is no doubt that APM reduces the energy density of the foods or beverages it is added to. However, there is interest in whether this reduction results in lower overall energy intake, body mass and adiposity [24]. Analysis of energy consumption in this study showed APM consuming animals to reduce their energy intake by ∼17 and 25% for lean and HF fed animals respectively. This reduction in energy intake occurred in spite of identical diet composition within groups, as APM was only administered in the drinking water. Reductions in energy intake with APM resulted in lower body mass in both CH and HF. In spite of this, there were discrepant effects of APM on body fat; the percentage increased in CHA, yet resulted in a lower body fat gain in HFA compared to their respective water consuming controls. To gain insight into the impact of these alterations on metabolic health, an OGTT and ITT were performed. Fasting hyperglycemia was evident with APM ingestion regardless of diet. Furthermore, the AUC for glucose during the ITT was elevated in APM rats in both the CH and HF diet conditions. By administering a high physiological insulin bolus, we were able to show a reduced ability of animals to clear endogenous glucose with APM, either due to a reduction in peripheral insulin sensitivity or an impaired insulin-mediated suppression of net hepatic glucose output. Given the OGTT results showed no difference with APM, the latter hypothesis is likely correct.

To explore the potential mechanism(s) by which APM affects metabolism, gut microbiota and serum metabolomics analyses were performed. Increasing evidence points to a significant interaction between the gut microbiome, the metabolomic profile and the development of metabolic disease states [27]. We found HFW, but not HFA, to be associated with a more obesity and diabetes-associated microbiota profile as defined by a higher Firmicutes:Bacteroidetes ratio. This indicates that APM treatment may have provided a protective effect against HF-induced changes in microbial phenotype, although this is likely a simplistic view given that high throughput sequencing now allows for greater insight down to the species level. However, APM also associated with an increased proportion of *Enterobacteriaceae* when combined with a HF diet. Members of the Proteobacteria phylum, including *Enterobacteriaceae*, produce gases and SCFA that have been previously associated with inflammation and insulin resistance [28]. Likewise, APM consumption in conjunction with HF also decreased *Clostridium* Cluster XI, from which pathogenic bacteria can arise. This cluster may also have contributed to the significant decreased in butyrate (**Figure 3**), as it contains many butyrate-producing bacteria.

It is well established that microbiota communicate and mediate many of their benefits to the host organism through a variety of secreted metabolites [27], [29]. Given this, serum metabolomics analysis was performed. Results demonstrated numerous serum metabolites changing in response to both diet and APM consumption, with the most predominant changes noted in the SCFA (**Table 3**). These metabolites are important as they represent the end products of bacterial fermentation and are key signaling intermediates between the microbiota and host [27], [29]. APM associated with changes in acetate and butyrate in CH fed, but not HF animals. In both CH and HF, APM, resulted in a particularly large elevations in propionate, greater than any other SCFA examined. This is likely attributable to increases in *Clostridium* that produces the metabolite during the fermentation of oligosaccharides [30].

Propionate is rapidly gaining recognition for its communicative role between gut bacteria and the host and has been implicated in altering gene expression [31], insulin resistance [32], behaviour [33], [34], overall metabolic health [27], taste aversion [35], irritable bowel syndrome [36] as well as mitochondrial dysfunction and autism [15], [37], [38]. Hence, there are multiple mechanisms and interactions that could explain the involvement of propionate with APM in the present study. In particular, the observed changes in insulin tolerance may be attributable to alterations in mitochondrial function, perhaps by impairing fatty acid metabolism [37]. Alternatively, propionate is known to impact on immune system [39], [40] colonic motility and permeability [27], [41], [42], functions that likely influence host gut microbiota. Applicable to the results of the present study, propionate has also been identified as a highly efficient gluconeogenic substrate for both the intestine and the liver [29]. Employing the intestinal G6P knockout mouse (I-G6pc-/-), work by De Vadder and colleagues [27] shows that the conversion of propionate via intestinal gluconeogenesis results in the release of glucose in to the portal circulation resulting in metabolic benefits to the host including the activation of G-coupled free fatty acid receptors FFAR2 and FFAR3 as well as GLP-1 secretion [43], [44]. However, when produced in the colon, propionate directly enters the entero-hepatic circulation, reaching the liver as propionate. At the liver, this SCFA undergoes gluconeogenesis, contributing to hepatic glucose production, resulting in a deterioration of both glucose and insulin tolerance in the I-G6pc-/- mouse [27].

Given the above-mentioned results showing changes in both gut microbiota and SCFA, it is hypothesized that APM alters gut microbiota to favor propionate production in the colon. The end result may be an elevation in hepatic gluconeogenesis and therefore an increase in net hepatic glucose output. This mechanism may explain the higher fasting glucose levels as well as the reduction in insulin-stimulated suppression of gluconeogenesis during the ITT observed in this study. The effects of APM on gluconeogenesis would also be amplified in the obese state, as there is resistance to the insulin-mediated suppression of liver gluconeogenesis [45]. In agreement, the non-nutritive sweetener D-tagatose has previously been found to elevate propionate levels in the lower large intestine of pigs [46].

From a dietary perspective, small amounts of APM as well as its decomposition products may reach the colon, influencing the gut microbiota. APM is quickly hydrolyzed in the intestine into methanol, phenylalanine, and aspartate, as previously mentioned [47]. The systemic concentrations of these metabolites are thought to remain unchanged post consumption based on studies underlying the statement that APM is considered safe at “current levels of exposure”, as stated by the European Food Safety Authority [4]. In agreement, our serum metabolomics analysis showed no difference in APM breakdown products between treatments, evidence of the small dose of APM ingested, and that the compound was rapidly metabolized and excreted by rats. Conversely, small amounts of APM have been detected in the feces of Rhesus monkeys after the administration of ∼20 mg/kg, a very large pharmacological dose [47]. Likewise, APM can also decompose to cyclo-Asp-Phe, ∝-Asp-Phe, and β-Asp-Phe [48]. Of these components, not all are absorbed in the small intestine, hence some make their way to the colon where they can be fermented by the gut microbiota [48]. The presence of such compounds could potentially explain the alterations in the gut microbiota seen in the APM animals. Of note, none of these di-peptides were detectable by the metabolomics method (^1^H NMR) employed in this study.

In summary, results of this study show APM to mitigate many of the negative effects associated with HF feeding including lower body mass, adiposity, caloric consumption and fasting insulin levels. In spite of this, APM resulted in hyperglycemia and an impaired ability to respond to insulin (ITT), which could be due to enhanced gluconeogenesis fueled by production of the SCFA propionate by the gut microbiota. This mechanism warrants future investigation and may explain the increased risk of metabolic disease states with regular APM consumption observed in population-based studies.

## Supporting Information

Figure S1 **Principal component analysis score scatterplot of the serum metabolome showing all four treatment groups.** The unsupervised multivariate statistical model showing how samples within each diet and fluid group cluster together based on their respective metabolic profiles. Each dot represents one individual rat based on serum metabolic profile. The axis represents the principal components (PC) with the explain variation in percentage indicated for each PC. The ellipse, representing the 95% confidence interval, is shown to facilitate visualization of outliers. Abbreviations are as follows: CHW, chow water; CHA, chow aspartame; HFW, high fat water; HFA, high fat aspartame.
(TIF)

Figure S2 **Principal component analysis score scatterplot of the serum metabolome showing individual comparisons.** The four unsupervised multivariate statistical models based on **Figure S1** showing comparisons associated with **A–B**. diet (chow vs. high fat) and **C–D**. fluid (water vs. aspartame) treatment. Each dot represents one individual rat based on the serum metabolic profile. The axis represents the principal components (PC) with the explain variation in percentage indicated for each PC. The ellipse, representing the 95% confidence interval, is shown to facilitate visualization of outliers. Abbreviations are as follows: CHW, chow water; CHA, chow aspartame; HFW, high fat water; HFA, high fat aspartame.
(TIF)
