# Supplementary figures and images for: Low-Dose Aspartame Consumption Differentially Affects Gut Microbiota-Host Metabolic Interactions in the Diet-Induced Obese Rat
Source: PLoS One. 2014 Oct 14;9(10):e109841. doi: 10.1371/journal.pone.0109841 (PMC4197030; doi:10.1371/journal.pone.0109841)

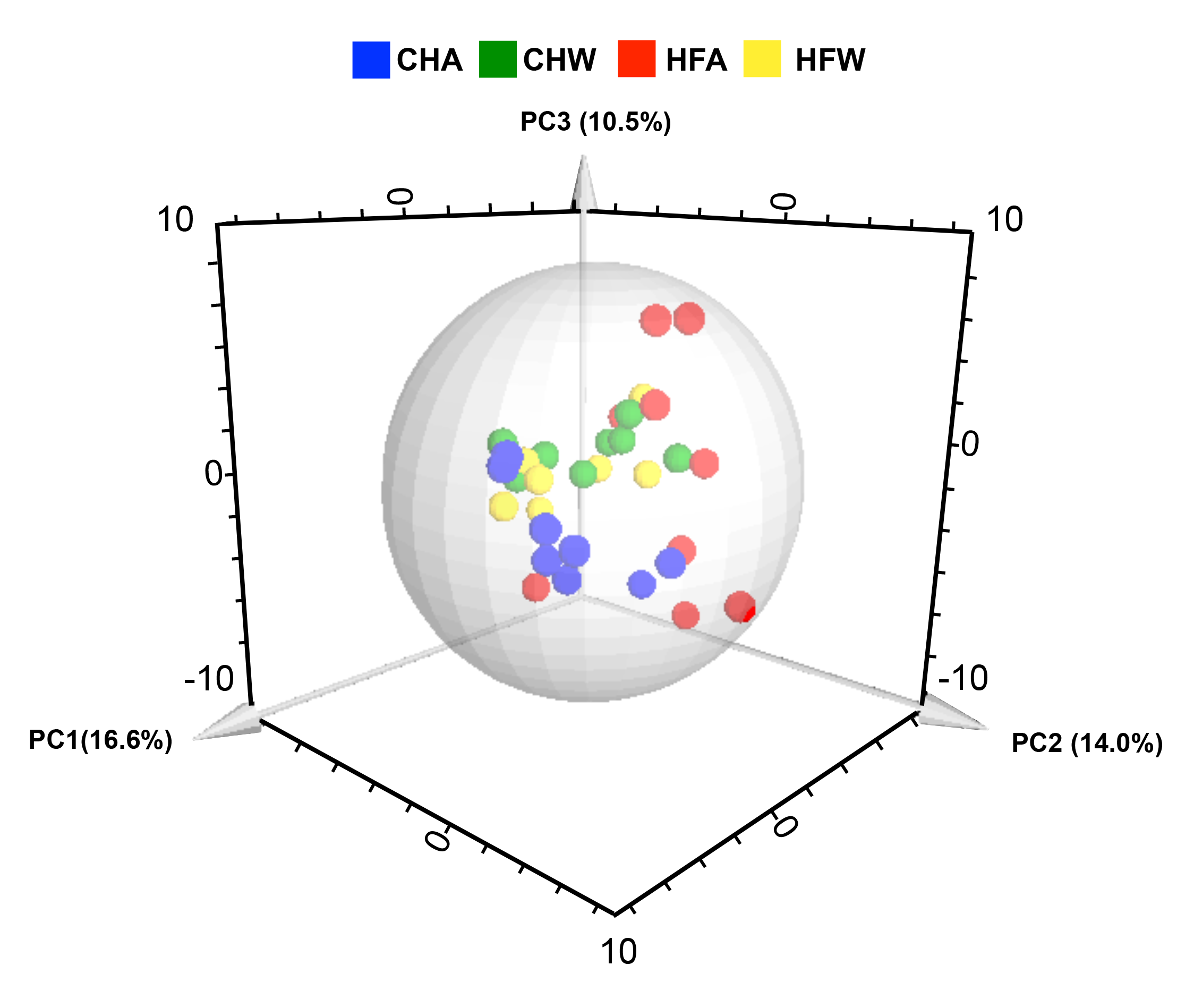

Supplement: Figure S1 — Principal component analysis score scatterplot of the serum metabolome showing all four treatment groups. The unsupervised multivariate statistical model showing how samples within each diet and fluid group cluster together based on their respective metabolic profiles. Each dot represents one individual rat based on serum metabolic profile. The axis represents the principal components (PC) with the explain variation in percentage indicated for each PC. The ellipse, representing the 95% confidence interval, is shown to facilitate visualization of outliers. Abbreviations are as follows: CHW, chow water; CHA, chow aspartame; HFW, high fat water; HFA, high fat aspartame. (TIF) [file pone.0109841.s001.tif]

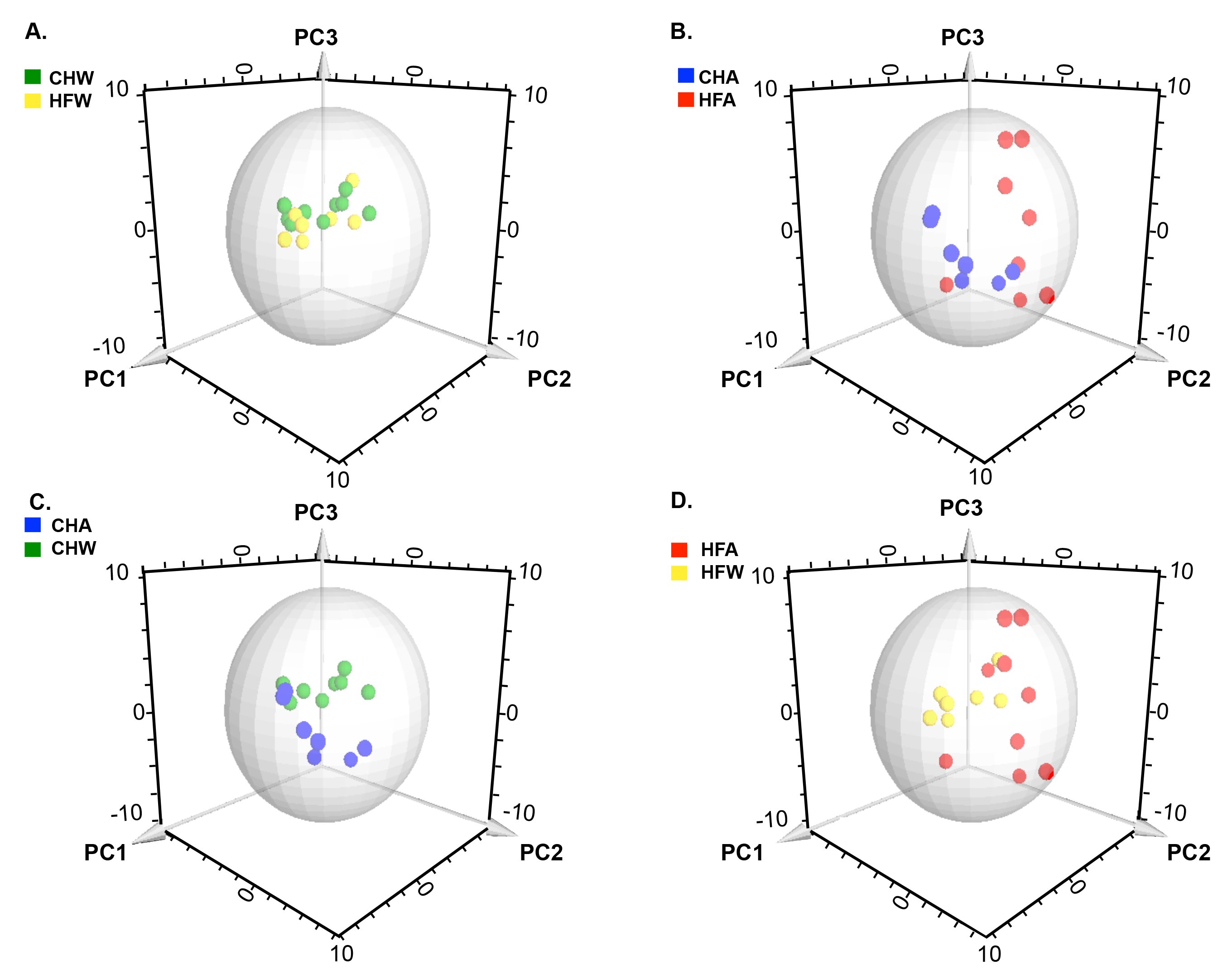

Supplement: Figure S2 — Principal component analysis score scatterplot of the serum metabolome showing individual comparisons. The four unsupervised multivariate statistical models based on Figure S1 showing comparisons associated with A–B. diet (chow vs. high fat) and C–D. fluid (water vs. aspartame) treatment. Each dot represents one individual rat based on the serum metabolic profile. The axis represents the principal components (PC) with the explain variation in percentage indicated for each PC. The ellipse, representing the 95% confidence interval, is shown to facilitate visualization of outliers. Abbreviations are as follows: CHW, chow water; CHA, chow aspartame; HFW, high fat water; HFA, high fat aspartame. (TIF) [file pone.0109841.s002.tif]
